# Spatial characterization of RPE structure and lipids in the PEX1-p.Gly844Asp mouse model for Zellweger spectrum disorder

**DOI:** 10.1016/j.jlr.2025.100771

**Published:** 2025-03-07

**Authors:** Samy Omri, Catherine Argyriou, Rachel S. Pryce, Erminia Di Pietro, Pierre Chaurand, Nancy Braverman

**Affiliations:** 1 Child Health and Human Development Axis, Research Institute of the McGill University Health Centre, Montréal, Québec, Canada; 2 Department of Human Genetics, McGill University, Montréal, Québec, Canada; 3 Department of Chemistry, Université de Montréal, Montréal, Québec, Canada

**Keywords:** dyslipidemia, eye/retina, inflammation, lipidomics, lipids, mass spectrometry imaging, peroxisome disease, *PEX1*, retinal degeneration, retinal pigment epithelium

## Abstract

Zellweger Spectrum Disorder (ZSD) is caused by defects in *PEX* genes, whose proteins are required for peroxisome assembly and function. Peroxisome dysfunction in ZSD causes multisystem effects, with progressive retinal degeneration (RD) among the most frequent clinical findings. However, much remains unknown about how peroxisome deficiency causes RD. To study RD pathophysiology in ZSD, we used the PEX1-p.Gly844Asp (G844D) mouse model, which represents the common human PEX1-p.Gly843Asp (G843D) variant. We previously reported diminished retinal function, diminished functional vision, and neural retina structural defects in this model. Here, we investigate the retinal pigment epithelium (RPE) phenotype, examining morphological, inflammatory, and lipid changes at 1, 3, and 6 months of age. We report that RPE cells exhibit evident degeneration by 3 months that worsens with time, starts in the dorsal pole, and is accompanied by subretinal inflammatory cell infiltration. We match these events with imaging mass spectrometry for regional analysis of lipids in the RPE. We identified 47 lipid alterations preceding structural changes, 9 of which localize to the dorsal pole. 29 of these persist to 3 months, with remodeling of the dorsal pole lipid signature. 13 new alterations occur concurrent with histological changes. Abnormalities in peroxisome-dependent lipids detected by LC/MS/MS are exacerbated over time. This study represents the first characterization of RPE in a ZSD model, and the first in situ lipid analysis in peroxisome-deficient tissue. Our findings uncover potential lipid drivers of RD progression in ZSD, and identify candidate biomarkers for retinopathy progression and response to therapy.

Peroxisome Biogenesis Disorders - Zellweger Spectrum Disorders (PBD-ZSD) are a heterogenous group of primarily autosomal recessive disorders due to defects in *PEX* genes whose protein products are required for peroxisome assembly and function (1, 2). Peroxisomes are ubiquitous organelles that play a critical role in complex lipid metabolism. Each peroxisome contains more than 50 enzymes whose functions include β-oxidation of very long chain fatty acids (VLCFA, carbon chains ≥ C22), the generation of the polyunsaturated fatty acid (PUFA) docosahexaenoic acid (DHA), and precursors for plasmalogen lipids (3). Dysfunctional peroxisomes in ZSD cause multisystem effects, with progressive retinal degeneration (RD) leading to childhood blindness being one of the most frequent clinical findings. There is currently no treatment for ZSD, and management is supportive only (4).

Despite significant progress in understanding the role of peroxisomes in normal cellular functions, much remains unknown about how their deficiency causes the clinical phenotypes. Thus, we generated a knock-in (PEX1-G844D) mouse model representing the common human *PEX1*-c.2528G>A allele (encoding PEX1-G843D) to study pathophysiology and develop therapies, and found that it has classic features of milder ZSD, including retinal degeneration (5). We previously showed that this model exhibits consistently diminished cone photoreceptor function, measured from 2-32 weeks of age, with rod photoreceptor function diminishing over time, and reduced visual acuity. In addition, this model exhibits diminished cone cell numbers by 6 weeks, decreased bipolar cell numbers with age (32 weeks), and disorganization of the photoreceptor inner segment ultrastructure (6, 7). These studies are consistent with the RD observed in patients with ZSD such as reduced visual acuity and cone anomalies (8).

Our previously reported ocular assessments of PEX1-G844D mice focused on retinal function and neural retina structure. However, retinal pigmentary changes are also commonly observed clinically in ZSD, suggesting the retinal pigment epithelium (RPE) is also affected in this disease (8). Moreover, a similar RD process to that of PEX1-G844D mice was described in the *Mfp2*^*−/−*^ mouse model, which is null for a major enzyme in peroxisomal β-oxidation of very-long-chain fatty acids (VLCFA). This model showed earlier involvement of photoreceptor and RPE layers (9). Selective inactivation of *Mfp2* in photoreceptors surprisingly did not result in their damage, suggesting that photoreceptor death in global *Mfp2*^*−/−*^ mice was not driven cell autonomously (10). In contrast, selective inactivation of *Mfp2* in RPE resulted in RPE degeneration and secondary photoreceptor death (11). Taken together, the observations in ZSD patients and *Mfp2*-deficient mouse models support the importance of peroxisome functions for RPE health. Here, we investigate the RPE phenotype in our PEX1-G844D mouse model for mild ZSD, observing morphological, inflammatory, and lipid changes over time. We report that RPE cell degeneration appears by 3 months of age and worsens with time, starts in the dorsal pole, and is accompanied by inflammatory activation. We match these events with lipid remodeling using mass spectrometry imaging (MSI) and liquid chromatography tandem mass spectrometry (LC/MS/MS), identifying the earliest lipid changes that precede structural alterations, as well as lipid profiles at later disease stages.

## Materials and Methods

### Mouse husbandry

*Pex1-c.2531G>A* (PEX1-G844D) heterozygous mice were maintained on congenic 129/SvEv (129S6.Cg-Pex1^tm1.1Sjms^/Mmjax) and C57BL/6N (B6.Cg-^Pex1tm1.1Sjms^/Mmjax) backgrounds. As >80% of PEX1-G844D homozygotes die before weaning on either congenic background (12, 13) all experiments were performed on mice with a stable mixed background of 50% C57Bl/6% and 50% 129 Sv/Ev. The C57Bl/6 strain used is negative for the Rd8 mutation of the *Crb1* gene. The Rd8 mutation was removed by crossing with the Crb1^cor^ C57BL/6N strain (JAX 022521). To minimize genetic variation, we used the F1 generation of two congenic PEX1-G844D heterozygote parents (♂C57Bl/6 x ♀129 Sv/Ev, or ♀C57Bl/6 x ♂129 Sv/Ev). We observed no effects of parental imprinting or sex. Nearly all (>90%) PEX1-G844D homozygous F1 progeny survive past weaning, after which we observe no effect of the mutation on life expectancy. Mice were housed at the RI-MUHC Glen site animal care facility with ad libitum access to food and water. All experiments were performed at the RI-MUHC Glen site and approved by the Research Institute of the McGill University Health Centre Animal Care Committee. Euthanasia was performed using CO_2_ under isoflurane anesthesia (5% isoflurane in oxygen until loss of consciousness, immediately followed by CO_2_ at maximum flow rate, 4 LPM). Both males and females were used for all experiments, and wild-type and PEX1-G844D heterozygous mice used as littermate controls. There were no phenotypic differences based on sex or control genotype. Genotyping was performed as previously described (6).

### Retinal flatmount preparation and immunofluorescence

Mouse eyes were enucleated and fixed in 4% formalin for 5 min at room temperature, at which point a hole was made in the cornea with a needle (27G), and eyes fixed for an additional 25 min. Fixed eyes were sectioned at the limbus and anterior segments discarded. The posterior eye cups consisting of the neural retina/RPE/choroid/sclera complex were collected and the neural retina was carefully detached from RPE/choroid/sclera to be prepared separately for experiments.

#### RPE/choroid/sclera flatmounts

Were treated with 0.1% Triton X-100 in phosphate buffered saline (PBS) for 45 min. Specimens were incubated overnight at 4°C with 1:400 TRITC phalloidin (ECM Biosciences, PF7551 Versailles, KY) for RPE actin cytoskeleton labeling. Primary antibody used was 1:400 rabbit anti-human IBA1 (Wako 019–19741) with secondary antibody 1:450 anti-rabbit Alexa Fluor 488 (Invitrogen A21206). Immunofluorescence was analyzed and images acquired using a Zeiss LSM780 laser scanning confocal microscope. We counted the number of RPE cells or IBA1 positive cells in eight fields of view spanning the four poles of each RPE flatmount (excluding the center, containing the optic nerve head) to quantify RPE degeneration or inflammation, respectively, and reported this as cells/m^2^.

#### Neural retina flatmounts

Were treated with saturation buffer (0.1% Triton X-100% and 10% fetal bovine serum (FBS) in PBS) for 45 min. Cone photoreceptors were stained overnight at 4°C with 1:500 fluorescein peanut agglutinin (Vector Laboratories) diluted in saturation buffer. Imaging was performed as above.

### Retinal immunohistochemistry

Eye cups from PBS-perfused mice were fixed 3 h in 4% formalin, incubated in 10% (30 min on ice), 20% (1 h on ice), and 30% (4°C overnight) sucrose in 0.1 M PB, then embedded and frozen in frozen section compound (VWR). 10 μm retinal cryo-sections were blocked 0.1% Triton X-100% and 10% FBS in PBS) for 1 h, washed, incubated at 4°C overnight with 1:400 rabbit anti-human GFAP (Abcam ab7260) in incubation buffer (0.1% Triton X-100, 10% FBS in PBS). Following primary antibody incubation, sections were washed and incubated 90 min with 1:450 anti-rabbit Alexa Fluor 594 (Invitrogen A21207) and washed again. Coverslips were mounted using ProLong Gold antifade reagent with DAPI (Invitrogen) and retinas were visualized by confocal microscopy as above.

### Matrix-assisted laser desorption ionization (MALDI) mass spectrometry

RPE was harvested from sets of three PEX1-G844D and WT mice at 30 and 90 days of age. The tissues were flat-mounted onto conductive indium tin oxide-coated microscope slides (Delta-Technologies Ltd. Loveland).

#### Silver-assisted laser desorption ionization (LDI) mass spectrometry imaging (MSI)

Silver-assisted LDI MSI was performed after silver sputter deposition for 30 s at 80 mA and 0.02 mbar of argon partial pressure on the RPE sections using a Cressington 308R sputter coater system (Ted Pella Inc.) as previously described (14). Sections were then analyzed in positive ion mode with reflectron geometry using an ultrafleXtreme MALDI-TOF mass spectrometer (Bruker Daltonics). Source parameters including delayed extraction and laser energy were optimized for maximum mass resolving power and signal to noise. Automated MSI data acquisition was performed at a spatial resolution of 150 μm averaging 300 shots per coordinates. MSI data acquisition was performed using the Bruker Daltonics flexImaging V4.1software.

#### Dual-polarity MALDI MSI

Dual-polarity MALDI MSI was performed after 1,5-diaminonapthlene matrix spraying using a first-generation TM-Sprayer (HTX Technologies). Matrix was sprayed at concentration of 10 mg/ml in 50:50 ACN:H_2_O, 0.1% trifluoracetic acid with the following protocol: 0.05 ml/min flow rate, 20 passes, 1200 mm/min track velocity, 4 mm track spacing with a 2 mm offset for even-number passes, a nozzle temperature of 40°C and an air pressure of 20 psi. The matrix thickness was calculated to be 0.21 mg/mm^2^. The tissues were then analyzed by MSI in reflectron mode under optimized ion source settings using the ultrafleXtreme system at a 150 μm spatial resolution averaging 150 laser shots in positive and 350 laser shots in negative ion per coordinate. The tissues were first imaged in positive ion mode. For negative ion mode MSI, the coordinate acquisition grid was offset by 75 μm in both the x and y directions allowing resampling of the same sections.

#### MSI data processing

MSI data processing was performed with the Cardinal V3 software within the R V4.2.3, environment along with some lab generated and base R functions (15). To determine the suitability of total ion current (TIC) normalization, the variance over the imaged area was calculated for the TIC of each slide, ranging from 25%-48%, which is similar to values obtained using homogenate tissues. Visual inspection of TIC distribution revealed no obvious bias towards either genotype or localization. Data was normalized by the total ion current and binned with a tolerance of 0.2 Da. For each considered *m/z* signal, average intensity values were generated per section or per RPE areas (dorsal and ventral). To reduce the effect of outlier pixels, the top and bottom 10% of the MSI data was not considered in the determination of mean values for each region.

#### Tandem mass spectrometry

MS/MS data acquisition for fatty acids and lipids was performed on a TimsTOF Flex mass spectrometer (Bruker Daltonics). Identifications based on exact mass only (mass accuracy < 2 ppm) were also determined using this instrument.

Lipid classification and nomenclature presented is based on that used by the LIPID MAPS Initiative (16, 17).

### LC/MS/MS analysis

For liquid chromatography-tandem mass spectrometry (LC/MS/MS) analysis, RPE was homogenized in PBS using a mini pestle. 2:1 chloroform/methanol containing 0.05% butylhydroxytoluene (BHT) was added to homogenized tissue in a glass tube and incubated on an orbital shaker at room temperature for 2 h. Samples were centrifuged at 2500 rpm for 10 min and the supernatant was transferred to a clean glass tube. The supernatant was washed with 0.2 volumes of purified water, mixed and centrifuged at 2000 rpm room temperature for 5 min to separate the two phases. The upper phase was removed and the lower phase washed with folch theoretical upper phase. (3:48:47 chloroform:methanol:water). The samples were mixed and centrifuged at 2000 rpm for 5 min. The upper phase was removed and the lower phase dried under nitrogen. The dried lipid was dissolved in 3:2 hexane:isopropanol containing 10 ng of each internal standard, 16:0-D4 lyso-PAF (20.6 pmol) and D4-26:0-lyso-PC (15.6 pmol). Samples were filtered by centrifugation through Costar spin-X centrifuge tube filters (Corning, NY) for 5 min. Filtrates were analyzed in Verex auto-sampler vials (Phenomenex, Torrance, CA). A 2.1 × 50 mm, 1.7 μm chromatography column and a Waters (Milford, MA) TQD (Triple Quadrupole Mass Spectrometer) interfaced with an Acquity UPLC (ultra-performance liquid chromatography) was used in positive ion electrospray (ESI)-MS/MS ionization. Solvent systems were: mobile phase A = 54.5% water/45% acetonitrile/0.5% formic acid, mobile phase B = 99.5% acetonitrile/0.5% formic acid with both solutions containing 2 mM ammonium formate. Initial solvent conditions were 85% mobile phase A/15% mobile phase B followed by a gradient from 15% to 100% mobile phase B over a period of 2.5 min, held at 100% mobile phase B for 1.5 min before reconditioning the column back to 85% mobile phase A/15% mobile phase B for 1 min at a solvent rate of 0.7 ml/min. A column temperature of 35°C and an injection volume of 5 μl was used for analysis. Ethanolamine plasmalogens were detected by multiple reaction monitoring (MRM) transitions representing fragmentation of [M+H]^+^ species to *m/z* 311, 339, 361, 385, 389 for compounds with 16:1, 18:1, 20:4. 22:6 and 22:4 at the sn-2 position, respectively. Lysophosphatidylcholine (LysoPC) species were detected by multiple reaction monitoring (MRM) transitions representing fragmentation of [M+H]^+^ species to *m/z* 104. Unlabeled standards of LPC and plasmalogen species were used to confirm precise and accurate detection of analytes. Semi-quantification was used to determine the amount of analyte by using the following formula (peak area analyte/peak area internal standard) x amount of internal standard. Reagents used were authentic plasmalogen standards, tetradeuterated internal standards 26:0-D4 lysoPC (Avanti Polar Lipids), 16:0-D4 lyso PAF (Cayman Chemical Company) and Optima grade solvents (methanol, acetonitrile, chloroform, water) (Fisher Scientific), formic acid (Honeywell Fluka), ammonium formate (Sigma-Aldrich), and PBS (Thermo Fisher Scientific).

Lipid classification and nomenclature presented is based on that used by the LIPID MAPS Initiative (16, 17).

### Statistical analysis

Results are expressed as mean ± standard deviation (SD). Comparisons of two groups were performed using unpaired Student’s *t* test, assuming normal distribution and homogeneity of variances. Comparisons between more than two groups were performed on normally distributed data using one-way ANOVA and Tukey’s multiple comparisons test. Statistical significance was set based on *P* value: ∗*P* < 0.05, ∗∗*P* < 0.01, ∗∗∗*P* < 0.001, ∗∗∗∗*P* < 0.0001. A minimum sample size of three was used for all experiments. Statistical analysis was performed using GraphPad Prism 10.0 software.

## Results

### Histological analysis of PEX1-G844D RPE and identification of early lipid changes

To investigate the initial stages of retinal changes in PEX1-G844D mice, we performed histological analyses in combination with mass spectrometry imaging (MSI) of the retinal pigment epithelium (RPE) at 1 month of age.

#### RPE morphology at 1 month

**Fig. 1:**
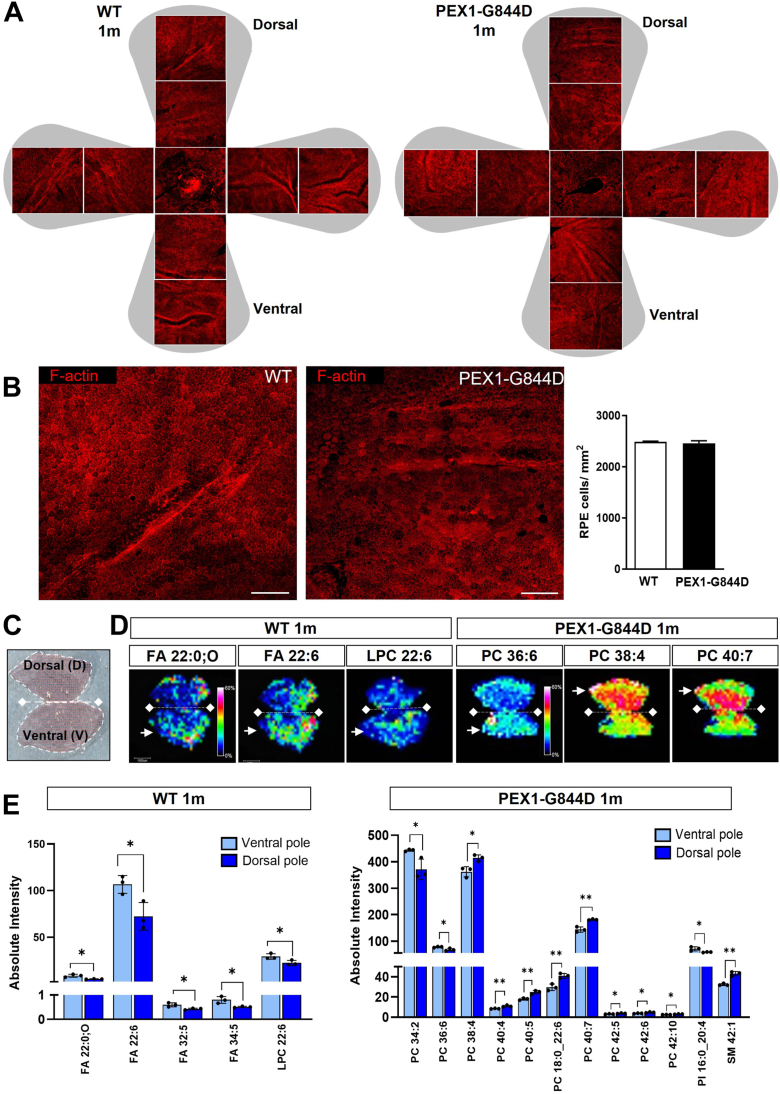
Structure and MALDI MSI lipid analyses on whole RPE flatmounts from 1-month-old PEX1-G844D mice. A: Confocal imaging of RPE flatmounts from 1-month-old WT and PEX1-G844D mice stained with the F-actin marker TRITC-phalloidin. The shaded grey area represents whole RPE tissue prepared for flat mounting. Nine confocal microscope images arranged in dorso-ventral orientation were taken at 20x magnification to visualize tissue integrity. B: Magnified representative RPE images are shown to facilitate observation of cell morphology; (scale bar: 100 μm). Histogram shows the quantification of RPE cell numbers from WT and PEX1-G844D RPE flatmounts (N = 3 per genotype). C: Image of the coordinate acquisition grid (red dots) for RPE sampling. D: Examples of MSI localization of lipids with heterogeneous spatial distribution (signal intensity). White arrows indicate the pole with the greatest abundance. E: Quantification of lipid abundance (signal intensity) in the dorsal and ventral poles. All lipids with different abundance (heterogeneous distribution) between the poles are shown. (N = 3 per genotype). ∗*P* < 0.05, ∗∗*P* < 0.01. Color scale indicates the lowest abundance in dark blue and highest in red/white.

We examined cell morphology in RPE flatmounts by labeling polymerized actin filaments (F-actin) with phalloidin-TRITC to visualize the cortical actin cytoskeleton delineating the RPE cells. Confocal imaging analysis revealed the characteristic “honeycomb” shape of the RPE in PEX1-G844D mice, with no apparent difference compared to littermate controls (Fig. 1A, B). We confirmed this by scoring the number of RPE cells in 8 fields of view spanning the four poles of each RPE flatmount (excluding the optic nerve area) and reported this as cells per surface area. Quantification confirmed normal integrity of the PEX1-G844D RPE layer with no significant lesions compared to age-matched control mice (Fig. 1B).

#### Early lipid changes in PEX1-G844D RPE at 1 month

In the absence of structural alterations to the RPE, we next examined the tissue for altered lipid composition. The presence of early RPE lipid changes could inform the pathophysiology of PEX1-G844D-induced photoreceptor dysfunction, given the importance of RPE membrane trafficking for photoreceptor support (18). Changes in membrane lipid composition can impact membrane fluidity and the function of membrane-bound proteins, which are crucial for nutrient transport, waste removal, and the visual cycle. Disruptions to lipid metabolism due to peroxisome deficiency in the RPE could thus lead to photoreceptor dysfunction and contribute to retinal disease.

To identify lipid changes in the PEX1-G844D RPE at 1 month of age, we used MSI, a technique relatively under-documented in retinal studies, especially with retinal flatmount preparations. MSI on flatmounted RPE instead of cryosections enabled more accurate and holistic visualization of lipid organization across the entire RPE, instead of only one section. Moreover, MSI enables lipid detection enriched for the RPE, with less contribution from the choroid and sclera that would be present in RPE tissues dissected for traditional liquid chromatography tandem mass spectrometry (LC/MSMS) analysis. We identified 72 lipids among phosphatidylcholines (PC), phosphatidylethanolamines (PE), phosphatidylinositol (PI), phosphatidyl acids (PA), and sphingomyelins (SM) (Supplemental Table S1). Precise lipid species identity was determined using MS/MS (Supplemental Table S2).

Unexpectedly, lipid profiling revealed a significant heterogeneous spatial distribution of five lipids (FA 22:0;O, FA 22:6, FA 32:5, FA 34:5, and LPC 22:6) in WT mice, all of which were more abundant in the ventral pole (Fig. 1C, D, Supplemental Table S3). This finding is novel and underscores the complexity of lipid metabolism within the retina under physiological conditions. The regional variability in lipid composition suggests that different areas of the RPE may have specialized functions, which could be critical for maintaining overall retinal health. In contrast to observations in WT tissues, MSI analysis of PEX1-G844D RPE revealed a significant heterogeneous distribution of a larger group of 12 lipids, all of which are distinct from those identified in controls: PC (34:2, and 36:6) and PI (16:0_20:4) were more abundant in the ventral pole, whereas PC (38:4, 40:4, 40:5 18:0_22:6, 40:7, 42:5, 42:6, 42:10) and SM 42:1 were more abundant in the dorsal pole (Fig. 1E).

**Fig. 2:**
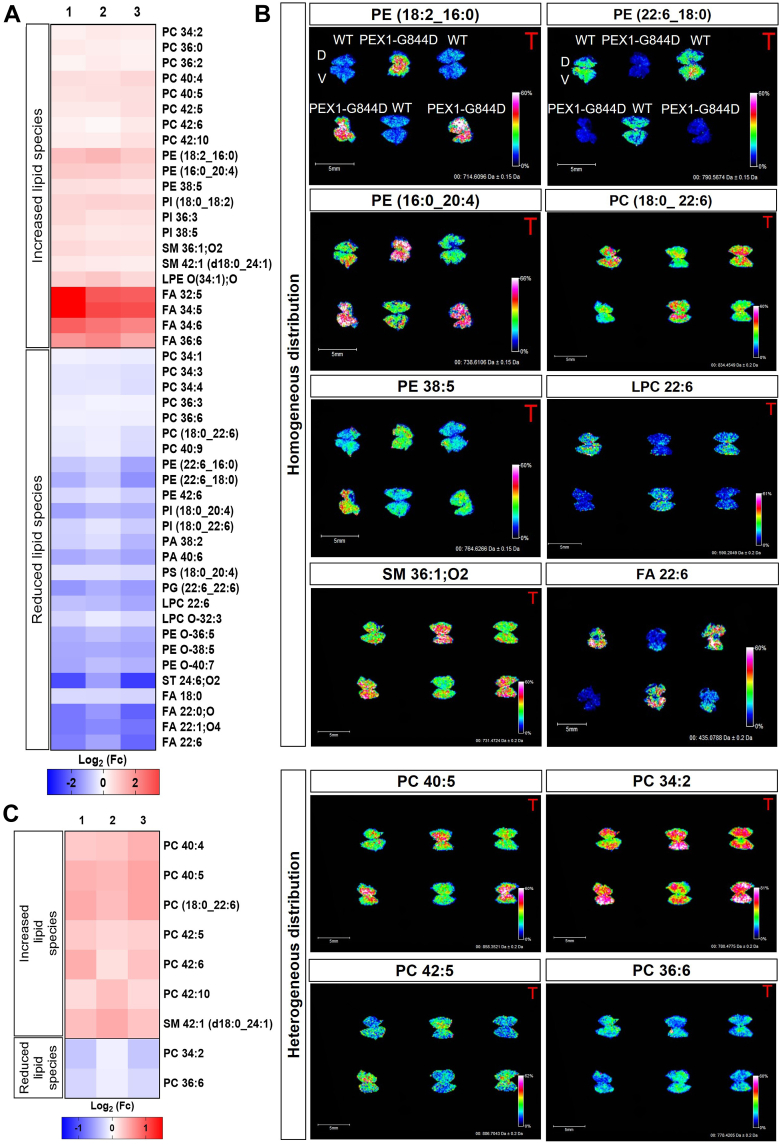
Early lipid changes in the PEX1-G844D RPE at 1 month of age. A: The heatmap shows the log_2_ fold change of lipid abundance in mutant samples compared to the WT average, with red indicating increased and blue decreased signal intensity. B: Localization by MALDI MSI of lipids in whole RPE flatmounts from WT and PEX1-G844D mice (6 RPE flatmounts per slide: 3 WT in V position and 3 PEX1-G844D in inverted V position). Examples MSI analyses show lipids with increased or reduced abundance in PEX1-G844D compared to controls, and homogeneous or heterogeneous distribution between the dorsal and ventral pole. The IMS scale indicates the highest lipid density in white/red and the lowest lipid density in dark blue. C: The heat map shows lipids dysregulated in PEX1-G844D with heterogeneous distribution between the dorsal compared to ventral pole (log_2_ fold change of lipid abundance). Only lipid species with statistically significant changes between mutant and WT RPE (Supplemental Table S1) are included in the heatmaps (A and C). Each column (1, 2, and 3) represents the ratio of signal intensity measured by MSI (A) in whole PEX1-G844D RPE compared to the average intensity in WT, or (C) in the PEX1-G844D dorsal pole compared to the PEX1-G844D ventral pole. Each row corresponds to one lipid species. (N = 3 per genotype).

The average relative abundance of all lipid species measured in whole RPE flatmounts from WT and PEX1-G844D mice is listed in Supplemental Table S1, along with the result of statistical comparison between the genotypes (*P*-value). These analyses revealed statistically significant differences in relative density in 47 lipids, presented in the heat map in Fig. 2A. MSI images illustrate examples of increased or reduced in mutant versus control RPE, with either homogeneous or heterogeneous distribution between the dorsal and ventral pole (Fig. 2B). Nine of these dysregulated lipids had different abundance between the dorsal and ventral pole exclusively in the mutants (Fig. 2C); PC 40:4, PC 40:5, PC 40:6 (18:0_22:6), PC 42:5, PC 42:6, PC 42:10, and SM 42:1 (d18:0_24:1) were increased, while PC 32:4 and PC 36:6 were decreased.

As expected with peroxisome dysfunction, our MSI analysis in PEX1-G844D RPE revealed a significant increase in very long-chain fatty acids (VLCFAs) including FA 34:5, FA 32:5, FA 34:6 and FA 36:6. We also observed a significant decrease in plasmalogens, such as LPC O-32:3, PE 16:0p20:4, PE O-38:5, PE O-40:7 and PA O-42:1. In addition, we detected a reduction in docosahexaenoic acid (C22:6, DHA) and multiple lipids with DHA incorporated, notably LPC 22:6, LPA 22:6, PC (18:0_ 22:6), PE (16:0_ 22:6), PE (18:0_ 22:6) PI (18:0_ 22:6) and PG (22:6_22:6). Overall, as the lipids identified in our analyses are primarily membrane-associated, our observations suggest that RPE membrane composition is altered in PEX1-G844D mice compared to wild-type controls, even at an early age and in the absence of structural defects.

### Structural changes follow lipid dysregulation in the PEX1-G844D retina

**Fig. 3:**
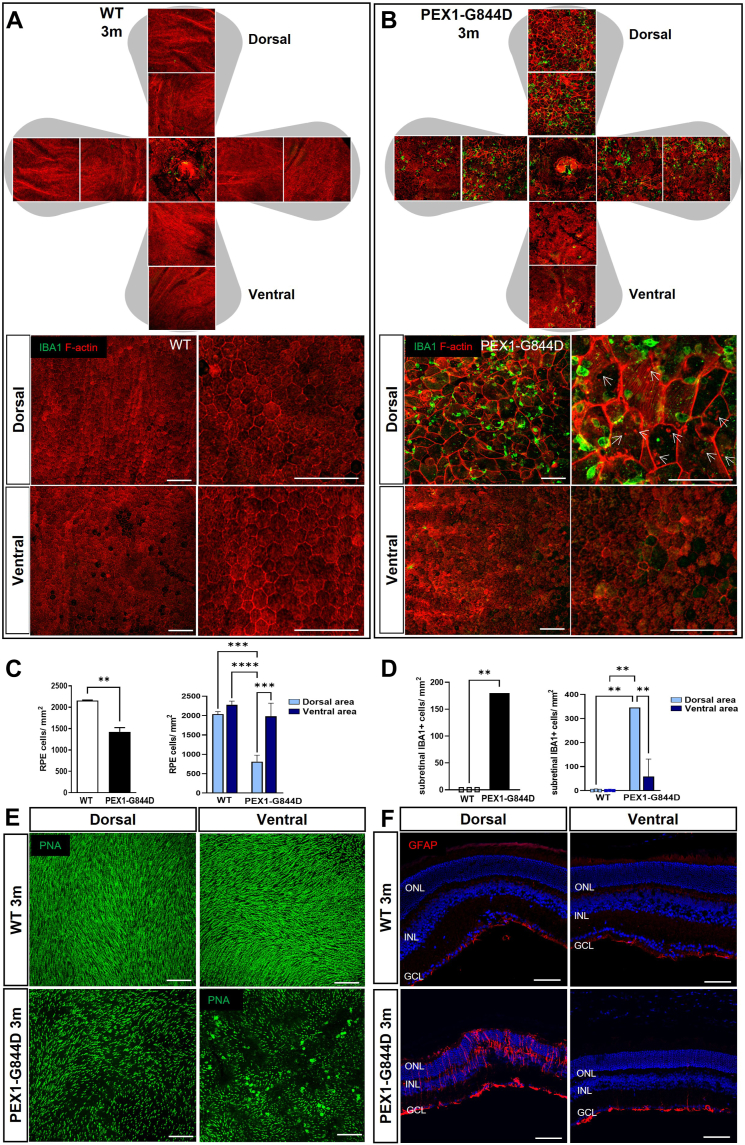
PEX1-G844D retinal structure at 3 months of age. A and B: Confocal imaging of RPE flatmounts from 3-month-old (A) WT and (B) PEX1-G844D mice immunostained with anti-IBA1 antibody, a microglia/macrophage marker (green) and counterstained with TRITC-phalloidin (red). Top panels: The shaded grey area represents whole RPE tissue prepared for flat mounting. Nine confocal microscope images arranged in dorso-ventral orientation were taken at 20x magnification to visualize tissue integrity. Bottom panels: Magnified representative images are shown to facilitate observation of cell morphology at dorsal and ventral poles. White arrows indicate blebs within RPE cells. C: Quantification of RPE cell number per mm^2^ in whole RPE tissue (left graph) and in dorso-ventral areas (right graph); N = 3 per genotype, ∗∗*P* < 0.01, ∗∗∗*P* < 0.001, ∗∗∗∗*P* < 0.0001. D: Quantification of subretinal IBA1 positive cells per mm^2^ on the whole RPE (left graph) and in dorso-ventral areas (right graph); N = 3 per genotype, ∗∗*P* < 0.01. E: Neuroretina flatmounts oriented with photoreceptors face up counterstained with the cone marker peanut agglutinin (PNA, green). F: Confocal imaging of dorsal and ventral poles of retinal cryosections, immunostained with anti-GFAP antibody (red) counterstained with DAPI (blue). GCL: ganglion cell layer; INL: inner nuclear layer; ONL: outer nuclear layer. Scale bar: 100 μm.

Our discovery of early lipid dysregulation in the PEX1-G844D mouse RPE prompted further investigation into the progression of RPE changes in this model. We expanded our histological study of RPE flatmounts to include tissues from 3-month-old mice. Staining of cortical actin filaments revealed profound structural changes in PEX1-G844D mice at this age (Fig. 3). Compared to WT control (Fig. 3A), PEX1-G844D RPE tissue exhibited profoundly enlarged cells with disorganized shape (Fig. 3B). This is a hallmark of RPE degeneration, wherein cell death results in enlargement of remaining cells to preserve the external blood retinal barrier function (19). Most strikingly, these structural abnormalities occurred mainly in the dorsal pole, where a subset of lipids was dysregulated at 1 month of age. Cell quantification showed a 35% reduction in the number of RPE cells per overall RPE surface area in PEX1-G844D compared to controls (Fig. 3C, left). In the dorsal area specifically, RPE cell density was reduced by 63% in PEX1-G844D mutants, while there was no difference between mutants and controls in the ventral area (Fig. 3C, right). In addition, we observed the presence of blebs on PEX1-G844D RPE plasma membranes (Fig. 3B, middle), indicative of RPE cellular stress (20).

Subretinal inflammation is a strong component of retinopathy progression in different RD models (age-related macular degeneration (AMD), diabetic retinopathy (DR)) (21, 22, 23, 24, 25), including other models of RD associated with lipid dysregulations such as the *Mfp2*^*−/−*^ and *Mfsd2a*^*−/−*^ mouse models (9, 26). Thus, we investigated whether the structural changes in PEX1-G844D RPE are associated with subretinal inflammation. Moreover, the presence or absence of inflammation and its impact on photoreceptors would allow for deeper understanding and characterization of the development of retinal disease in this model. Immunofluorescent microscopy of RPE flatmounts incubated with anti- Ionized calcium binding adaptor molecule 1a (IBA1, a marker of microglia/macrophages), showed infiltration of inflammatory cells in the PEX1-G844D subretinal space, indicating an active inflammatory response (Fig. 3A, B). On average, there were 175 IBA1 positive cells per mm^2^ in the PEX1-G844D RPE, and complete absence of these inflammatory cells in control tissues (Fig. 3D, left). Quantification by geographic area showed an accumulation of IBA1 positive cells mainly in the dorsal pole (300 cells/mm^2^) compared to the ventral pole (50 cells/mm^2^) of the retinal tissue (Fig. 3D, right). In parallel, the histological examination of the photoreceptor side of neuroretina flatmounts stained with a marker of cone extracellular matrix (peanut agglutinin, PNA) revealed decreased cone outer segment density. This occurred most prominently at the dorsal pole, in the same area as, and facing, the damaged RPE (Fig. 3E). Furthermore, immunofluorescent analyses of retinal cryosections showed increased levels of glial fibrillary acidic protein (GFAP), an indicator of gliosis, only in the dorsal pole (Fig. 3F). This indicates an acute retinal stress condition specifically in the dorsal zone of the PEX1-G844D retina.

### Correlation of structural alterations with RPE lipid dysregulation

**Fig. 4:**
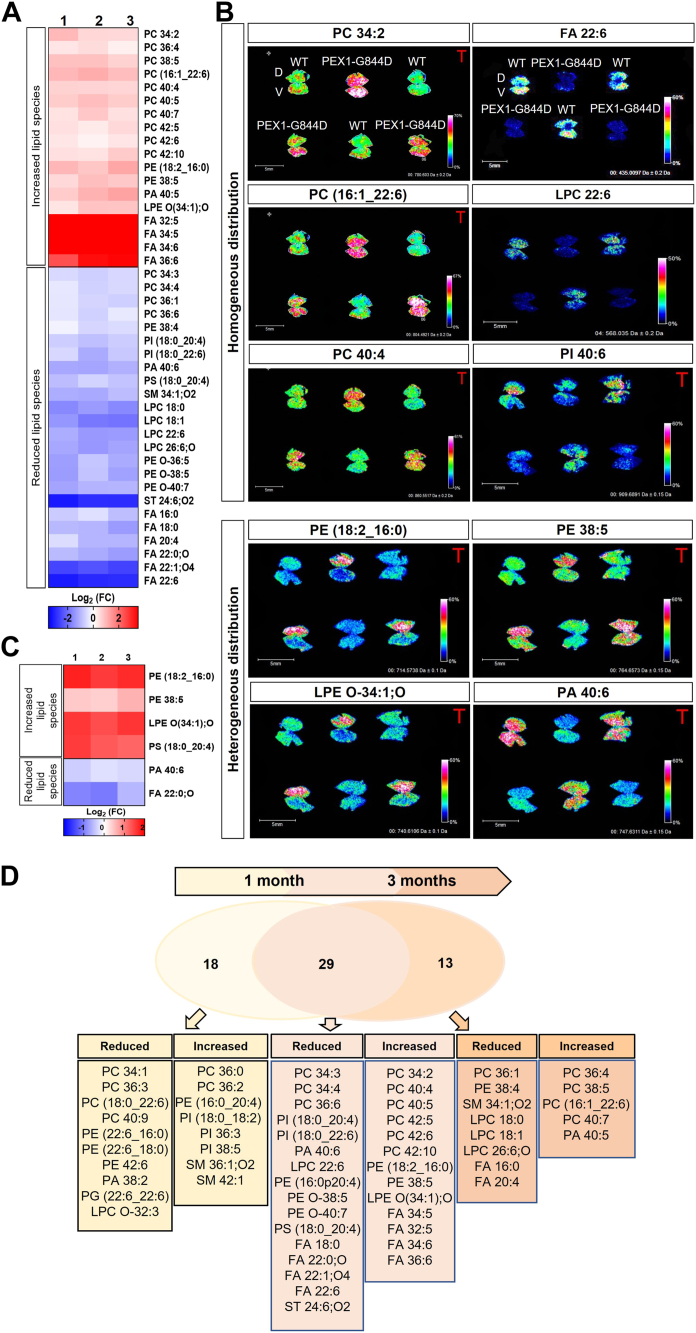
Progression of the spatial lipid changes in the PEX1-G844D RPE at 3 months. A: The heatmap shows the log_2_ fold change of signal intensity in mutant samples compared to the average intensity in WT samples, with red indicating increased intensity and blue indicating decreased intensity. B: Localization by MALDI MSI of lipids in whole RPE flatmounts from WT and PEX1-G844D mice (6 RPE flatmounts from 3-month-old mice: 3 WT in V position and 3 PEX1-G844D in inverted V position). Examples of MSI analyses showing 10 lipids identified with different abundance (homogeneous or heterogeneous) in PEX1-G844D RPE compared to WT control. C: The heat map shows the log_2_ fold change of lipid intensity in the PEX1-G844D dorsal compared to ventral pole. Only lipid species with significant difference between mutant and WT RPE (from Supplemental Table S4) are included in the heatmaps (A and C). Each column (1, 2, and 3) represents the ratio of signal intensity measured by IMS in (A) PEX1-G844D RPE samples compared to WT, or (C) PEX1-G844D dorsal compared to ventral pole. Each row corresponds to a lipid species. (N = 3 per genotype).The heat map color scales indicate highest lipid density in dark red and lowest lipid density in dark blue. D: The Venn diagram shows single lipid species significantly affected in PEX1-G844D mouse RPE at 1 and 3 months of age.

Our findings suggest that lipid dysregulation at earlier stages (by 1 month) may contribute to the observed inflammation and retinal damage at 3 months of age in PEX1-G844D mice. In this context, we analyzed RPE lipid distribution at 3 months of age using MSI, within this newly characterized inflammatory environment. Lipid profiling in 3-month-old RPE using MSI showed further changes in lipid composition than at 1 month, quantified and referenced in Supplemental Table S4. 42 lipid species showed statistically significant changes in PEX1-G844D RPE compared to controls (Fig. 4A). Among the twenty most abundant lipid species detected by MSI, 5 showed increased density in mutants: PC 34:2, PC 36:4, PC (16:1_22:6), PC 40:7 and PC 38:5, whereas three showed decreased density: PC 34:1, PC 36:1, PI (18:0_20:4) and SM 34:1; O2, suggesting a trend towards increasing polyunsaturated fatty acids. In situ examples of this differential lipid expression in PEX1-G844D and control RPE are illustrated in Fig. 4B, with increased lipid species presented in the left column, and reduced lipid species in the right column.

Most of the homogenous lipid changes observed in the PEX1-G844D RPE at 1 month persisted to 3 months. These included a further decrease of DHA and DHA-associated lipids (LPC 22:6, PI (18:0_22:6), and an exacerbated increase in VLCFAs (FA 32:5, FA 34:5, FA 34:6 and FA 36:6). These results indicated a sustained, and progressive, increase of polyunsaturated lipid species in PEX1-G844D RPE. Three of the lipid species that were elevated evenly throughout the RPE at 1 month showed a higher accumulation in the dorsal pole at 3 months: PE (18:2_16:0), PE 38:5, and LPE O-34:1; O (Fig. 4C, Supplemental Table S5). These lipids, which increase at the site of histological changes at 3 months, could be markers of or involved in active tissue damage and inflammation. Interestingly, the lipids previously elevated in the dorsal pole at 1 month were still increased compared to controls at 3 months (except for SM 42:1) but spread homogeneously throughout the whole RPE tissue. Early accumulation in the dorsal pole followed by a more generalized increase indicates that these lipids may accompany or follow the spread of tissue damage and inflammation. None of the lipids altered exclusively in the PEX1-G844D RPE dorsal pole at 1 month (Fig. 2C) overlapped those at 3 months (Fig. 4C).

We sorted the dysregulated lipids of the PEX1-G844D RPE by age (Fig. 4D). The levels of 47 lipids were precociously affected during the first month. 18 of them were modified only at early stages of the disease, before the onset of structural damage. The intersections of the diagram show that levels of 29 lipids were significantly modified and persisted over the first 3 months in PEX1-G844D RPE. These include the lipid species specifically elevated in the dorsal pole at 1 and 3 months. Compared to the whole RPE levels in WT, an additional 13 lipids showed significant changes in PEX1-G844D only once the structural phenotype appeared. In addition, 16 lipids showed differential dorso-ventral distribution in 3-month-old mutants, but not in WT. Eleven of these were relatively increased in the dorsal pole: PE (18:2_16:0), PE (16:0_20:4), PE 38:5, PI (18:0_18:2), PI (16:0_20:4), PS (18:0_20:4), LPE O(34:1);O, LPE O(34:0);O, LPC O-32:3, PA O-42:1, PA O-42:2, while five were decreased: PE (22:6_16:0), PE (22:6_18:0), PA 40:6, FA 22:0;O, FA 22:1;O4 (Supplemental Table S5).

**Fig. 5:**
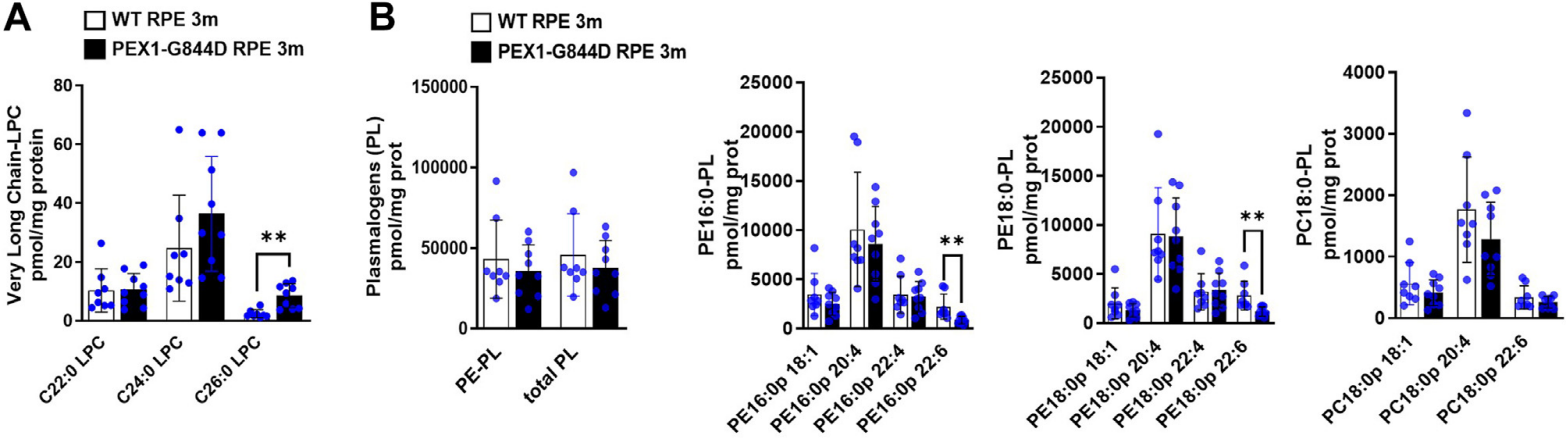
LC/MS/MS analyses of peroxisome metabolites at 3 months. Histograms present the levels of (A) very long chain-lysophosphatidylcholines (VLC-LPCs), and (B) phosphatidylethanolamine (PE), and phosphatidylcholine (PC) plasmalogens (PL) measured by LC/MS/MS in RPE tissue from WT and PEX1-G844D mice. (Each point represents 1 RPE; N = 8 per genotype, ∗∗*P* < 0.01).

Our MSI data revealed significant alterations in the lipid composition of the RPE membrane, associated with the development of RPE inflammation and lesions. We aimed to determine whether these findings could be associated with the presence of lipid biomarkers used in the clinical diagnosis of ZSD and detected by LC/MS/MS. Our LC/MSMS analysis of very long chain LPCs and PE-, PC-plasmalogens (PE-PLs, PC-PLs) in the isolated RPE-choroid-sclera complex revealed a 3-fold increase in C26:0 LPC levels in PEX1-G844D tissues (Fig. 5A), while overall (total) plasmalogen levels were not significantly altered. In contrast, a specific analysis of DHA-containing plasmalogens (C22:6) showed a 50% decrease of PE 16:0p22:6 and PE 18:0p22:6 (Fig. 5B). This observation corroborates the MSI analyses, which showed a consistent decrease in DHA-associated lipids. This complementary approach confirmed the simultaneous presence of ZSD biomarkers with the lipid changes revealed by MSI and the retinopathy phenotype.

### Structural defects extend to the entire PEX1-G844D RPE by 6 months

**Fig. 6:**
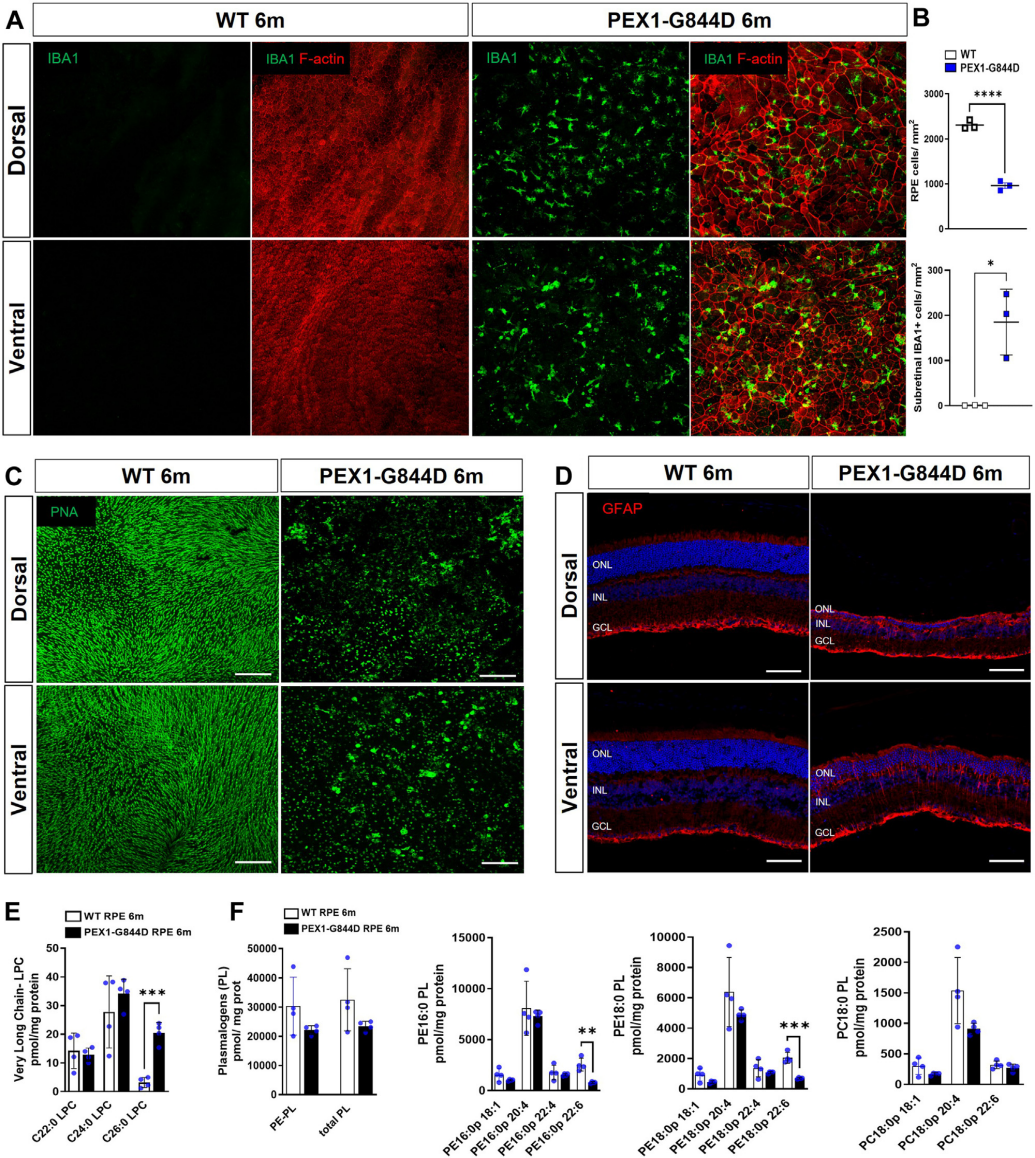
Later stage PEX1-G844D retinopathy phenotype at 6 months. A: Confocal imaging of dorsal and ventral areas of 6 month-old WT and PEX1-G844D RPE flatmounts immunostained with anti-IBA1 antibody, a microglia/macrophage marker (green) and counterstained with TRITC-phalloidin (red). B: Quantification of RPE cell number per mm^2^ in whole RPE tissue (top graph) and quantification of subretinal IBA1 positive cells per mm^2^ on the whole RPE (bottom graph, each point represents 1 RPE) (N = 3 per genotype, ∗*P* < 0.05, ∗∗∗∗*P* < 0.0001). C: Neuroretina flatmounts oriented with photoreceptors face up counterstained with peanut agglutinin (PNA), a cone marker (green). D: Confocal imaging of retinal cryosections from dorsal and ventral poles, immunostained with anti-GFAP antibody (red) counterstained with DAPI (blue). GCL: ganglion cell layer; INL: inner nuclear layer; ONL: outer nuclear layer. Scale bar: 100 μm. E, F: Histograms presenting the levels of (E) VLC-LPCs and (F) PE, PC plasmalogens (PL) measured by LC/MS/MS in RPE. (N = 4 per genotype, each point represents 1 RPE ∗∗*P* < 0.01, ∗∗∗*P* < 0.001).

To understand the long-term progression of inflammation and tissue damage in the PEX1-G844D mouse model, we extended histological analysis of the RPE and neural retina to 6 months. In addition, these studies would show whether the lipid changes that progressed from the dorsal to ventral pole at 3 months also preceded inflammation and tissue damage. Indeed, RPE flatmounts at 6 months of age demonstrated structural changes with enlarged and disorganized cells accompanied by subretinal inflammation throughout the entire RPE tissue (F-actin and IBA1 staining, Fig. 6A). Quantification of these changes showed a 60% reduction of RPE cells in PEX1-G844D compared to control mice, and an average of 180 IBA1 positive cells per mm^2^ (Fig. 6B). This is consistent with spatial lipid changes preceding structural events. Matching the widespread RPE degeneration, cone outer segment density (visualized using PNA) declined across the neural retina (Fig. 6C). Pan-retina photoreceptor cell loss occurred by this stage, evidenced by a thinning of the outer nuclear layer (ONL), although this was far more pronounced in the dorsal pole (Fig. 6D). At 6 months, gliosis (GFAP) was present in both dorsal and ventral poles (Fig. 6D). Changes in peroxisome biochemical metabolites in the PEX1-G844D RPE-choroid-sclera complex were exacerbated from 3 to 6 months; LC-MSMS analysis showed a 10-fold increase in C26:0 LPC levels in the RPE-choroid-sclera complex (Fig. 6E), and a 65% decrease in the DHA-containing plasmalogens PE 16:0p22:6 and PE 18:0p22:6 (Fig. 6F). Total plasmalogen levels remained normal, as did PC18:0p22:6.

### Comparison of PEX1-G844D-associated lipid changes with other RD mouse models

**Fig. 7:**
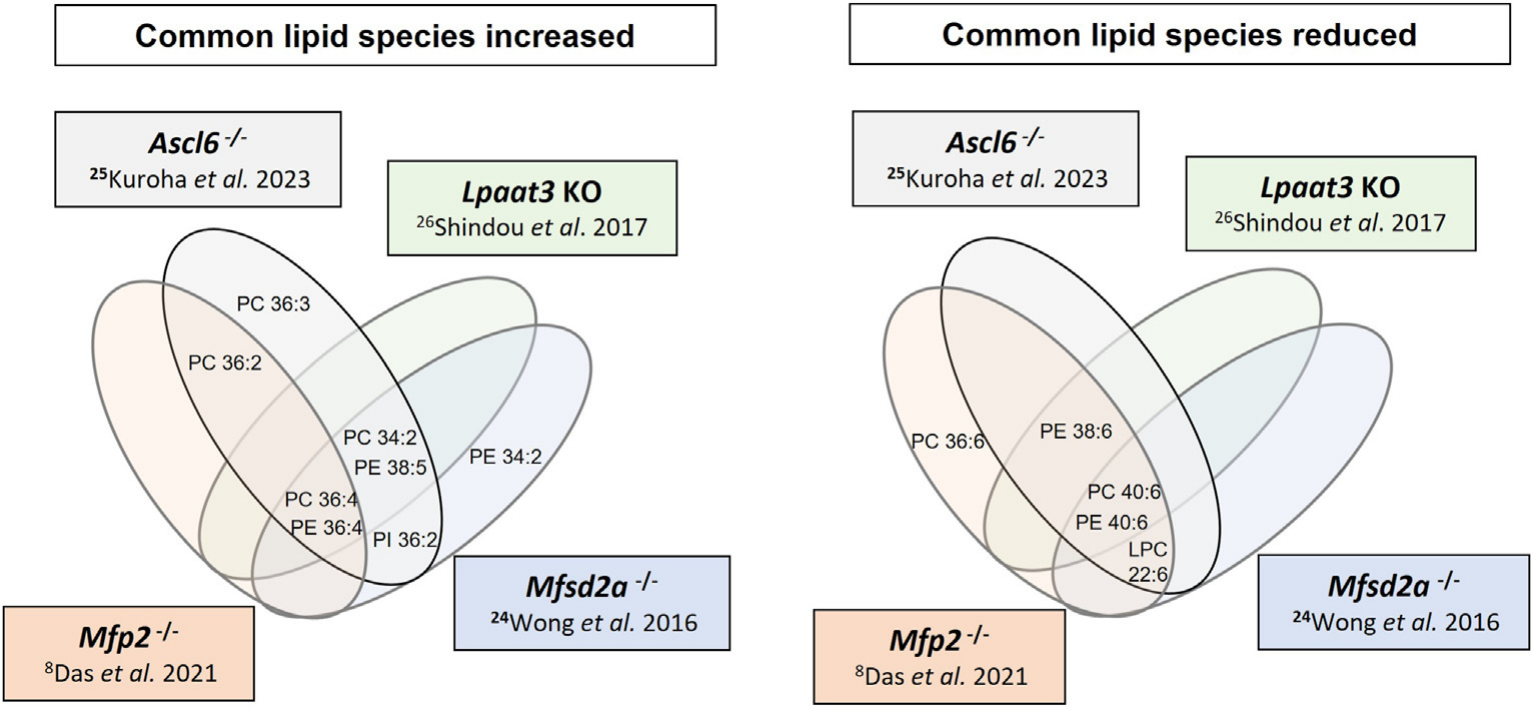
Comparison of common dysregulated lipids in PEX1-G844D RPE and other RD models. Venn diagrams comparing the overlap of lipid species affected in the PEX1-G844D RPE and in retinal tissue from other genetic RD mouse models with lipid dysregulation (*Ascl6*^*−/−*^, *Lpaat3*^*−/−*^, *Mfsd2a*^*−/−*^and *Mfp2*^*−/−*^). The diagram includes common lipids increased (left), and reduced (right) per model.

To understand whether the lipid changes observed in the RPE of PEX1-G844D mice could be directly linked to retinopathy progression, we investigated whether the lipids we identified by MSI in our model had already been described in different mouse models of RD. We identified from the scientific literature four genetically modified mouse models with altered lipids and RD: *Ascl6*^*−/−*^ (27), *Lpaat3*^−/−^ (28), *Mfp2*^*−/−*^ (9), and *Mfsd2a*^*−/−*^ (26). Lipidomic analyses from these models showed 13 dysregulated lipids in common with those we identified using MSI in PEX1-G844D RPE (Venn diagram, Fig. 7). Notably, increases in PC 36:4, PE 36:4 (16:0_20:4), PC 34:2, and PE 38:5 was observed in all models except *Mfp2*^*−/−*^, where PC 34:2 and PE 38:5 were not analyzed. Similarly, decreases in DHA-associated lipids, PC 40:6 (18:0_22:6), PE 40:6 (18:0_22:6), and LPC 22:6 was observed in all RD models except *LPAAT3*^*−/−*^, where LPC 22:6 was not analyzed. These lipid changes highlight a potential lipid signature associated with RD.

## Discussion

*PEX1*-associated Zellweger spectrum disorder (ZSD) results in peroxisomal dysfunction, leading to significant alterations in lipid metabolism. These changes are detectable at early disease stage and may thus contribute to disease pathogenesis and progression. In this study, we investigated the impact of peroxisome dysfunction on lipid metabolism and its subsequent effects on RPE structure in the PEX1-G844D mouse model of mild ZSD. This is the first report of large-scale lipid analysis using MSI on retinal tissue. This approach revealed significant early alterations in RPE lipid composition preceding the onset of structural changes, as well as distinct regional lipid profiles within the tissue. Furthermore, this study is the first to document subretinal inflammatory cells and RPE histology changes over time in any ZSD model, providing a more comprehensive understanding of the retinal phenotype in this disease.

### RPE lipid remodeling

Our findings highlight complex lipid dysregulation in the PEX1-G844D RPE, with 60 distinct lipids altered early in the disease process, showing increased or decreased abundance, and some exhibiting abnormal heterogeneous spatial distribution compared to littermate controls. Our results are consistent with the hallmark lipid abnormalities in ZSD patients, including elevated VLCFA levels, and decreased plasmalogens and DHA-associated lipids (4). In the context of retinal health, the reduction of DHA-associated lipids is particularly relevant, as DHA is essential for neuronal development and the maintenance of photoreceptor integrity and function, and is protective against oxidative stress (29, 30). Finally, DHA deficiency is a major contributor to RD in the *Mfp2*^*−/−*^ mouse model of peroxisome β-oxidation deficiency (31). As the RPE supplies DHA to photoreceptors, the early reduction in DHA-containing lipids in our PEX1-G844D mouse model likely contributes to the observed photoreceptor dysfunction and progressive structural degeneration.

### Role of inflammatory lipids

We identified alterations in PE lipids, including PE 16:0_20:4, PE 36:4, PE 18:2_16:0, and PE 38:5 which are associated with inflammatory environments. Elevated levels of PE 16:0_20:4 and PE 18:2_16:0 have been highlighted in studies on critical illness and inflammation (32), and Vianello *et al.* (2024) suggest that circulating PEs including PE 34:2, PE 38:5 are associated with the DAMP-induced NLRP3 inflammasome in cardiovascular disease patients with insulin resistance risk (33). In addition, PE 36:4 and PE 38:5 are reportedly elevated in the hippocampus of mice exposed to hypoxia, which causes inflammation (34). Moreover, our findings also include changes in PC lipids, including PC 36:2 and PC 36:4. These PCs are elevated in TNFα-treated HepG2 cells (35), suggesting association with inflammation. Our MSI results in the PEX1-G844D RPE underscore the broad impact of *Pex1* deficiency on lipid metabolism and its potential role in driving inflammation and retinal degeneration. This is supported by our histological study of retinal tissue including RPE at 3 and 6 months of age revealing increasing inflammatory cell infiltration over time, indicating an active inflammatory response. This is concurrent with profound structural changes featuring enlarged and disorganized RPE cells, starting mainly in the dorsal pole and extending to the entire tissue over time. In parallel, the observations of neural retina flat mounts and retinal cross-sections revealed decreased density of cone outer segments and outer nuclear layer thickness at 6 months, highlighting the consequences of chronic metabolic stress in the PEX1-G844D outer retina. Our data present for the first time in a ZSD mouse model a retinopathy phenotype dramatically affecting both the RPE and photoreceptor cells, with an inflammatory component. These results are consistent with the retinopathy phenotypes described in other models of RD associated with lipid dysregulation (9, 26, 27, 28).

### RPE regional changes

Our novel discovery of distinct lipid changes exclusively in the PEX1-G844D RPE dorsal pole suggests regional variations in lipid metabolism that correlate to subsequent structural damage. These lipids might be inherently toxic and contribute to cellular stress and inflammation, or this accumulation could represent a protective or compensatory response to initial cellular insult. Globally, elevated VLCFAs in the PEX1-G844D model can disrupt membrane integrity and induce oxidative stress, exacerbating retinal degeneration. Conversely, lipid accumulation might sequester harmful metabolites or provide an energy reserve during increased metabolic demand. In parallel, the reduction of some lipids in the dorsal pole before the onset of structural changes could indicate a lack of protective elements, exacerbating cellular stress. For instance, deficiency of DHA or other lipids might compromise cellular membrane integrity and the ability to respond to oxidative stress, making the RPE and photoreceptor cells more vulnerable to damage.

In control mice, the spatial heterogeneity of lipid distribution observed could indicate region-specific stress, functions, or metabolic need, and guide further research into regional variations within the RPE. One possible explanation for the lipid imbalance in the dorsal pole of the RPE may be the higher metabolic activity of M cones in this region. M cones, which are more abundant in the dorsal retina (36, 37, 38), have increased energy requirements relative to other cones (39), and therefore a higher rate of lipid consumption. In PEX1-G844D mice, this combination of higher metabolic need may cause this region, and the neighboring RPE, to be most sensitive to peroxisome deficiency and degenerate first. Studies of RPE cell population heterogeneity have explored topographic heterogeneity by comparing various cellular features (size, shape, and density) to uncover regional differences (40). Recently, emphasis on regional heterogeneity of RNA expression in RPE cells has been investigated (41). Our investigation of the spatial distribution of lipids within the *Pex1*-deficient RPE contributes to understanding how genetic mutations and regional metabolic activity affect lipid patterns and the tissue’s vulnerability to damage and disease progression. Lipid profiling by MSI in the PEX1-G844D RPE at 3 months compared to 1 month revealed further changes in lipid composition, a persistent decrease in DHA and its associated lipids, and an exacerbated increase in VLCFAs. In addition, the change, and increase, in lipids with unequal dorso-ventral distribution could result from an inflammatory environment. Overall, while the observed lipid changes are associated with PEX1-G844D-induced retinopathy, it is unknown whether they are related to the onset or progression of this disorder, or whether they are consequent to disease pathology. Regardless, the lipid changes identified when tissues are damaged could present a lipid signature of PEX1-G844D-induced retinopathy and be candidate biomarkers if detectable in blood or vitreous humor. Lipid measures are possible in human vitreous humor (42), with dysregulations reported in patients with diabetic retinopathy (43), including highly enriched polyunsaturated fatty acids linked to defects inflammation homeostasis (44).

### Comparison to lipid changes reported in other murine models of RD

To understand whether the lipid changes observed in the RPE of PEX1-G844D mice could be driving retinopathy progression, we compared our results with those of different RD mouse models. A literature comparison revealed four genetically modified mouse models (*Ascl*^*−/−*^, *Lpaat3*^−/−^, *Mfp2*^*−/−*^ and *Mfsd2a*^−/−^) with altered lipids and RD. Among these models, lipidomic analysis of retinal tissue revealed that each had between 7 and 11 lipids in common with the PEX1-G844D RPE MSI data. Notably, this included an increase in PC 34:2, PC 36:4, PE 36:4, and PE 38:5, as well as a decrease in DHA-associated lipids, observed in at least 3 out of 4 models. This commonality suggests that these lipids could be potential biomarkers or drivers for inflammatory conditions and/or retinopathy progression. Of note is that this list is underestimated insofar as not all lipids identified in our model were analyzed in each of the studies.

In addition to lipid dysregulation, we observed the formation of multiple vesicles or blebs accompanying the pronounced RPE actin cytoskeleton remodeling. Extracellular vesicles (EVs), which facilitate intercellular communication by transferring lipids, genetic material, and proteins, may contribute to the propagation of lipid imbalances and inflammatory signals, exacerbating retinal degeneration (20). Blebs, indicative of cellular stress, reflect the cellular response to lipid imbalance and structural changes in the RPE (45). If detectable in liquid biopsy from ZSD patients, RPE-derived EVs and blebs could serve as biomarkers for monitoring disease progression and therapeutic response.

### Novelty and limitations of the MSI approach

This study represents the first application of large-scale MSI on retinal tissue to investigate lipid metabolism changes in a peroxisome-deficient mouse model. This approach enabled us to map the spatial distribution of lipids with high resolution and sensitivity, uncovering significant early alterations in lipid composition preceding structural changes. The use of flatmounts allowed for a holistic visualization of lipid distribution across the entire RPE, providing a more comprehensive view compared to traditional sagittal retinal sections. The ability to visualize lipid alterations in a discrete cell layer provides a more precise observation of the metabolic landscape in a diseased tissue, which traditional lipidomic techniques cannot achieve. By employing MSI, we were able to identify distinct regional lipid profiles within the RPE and correlate them with structural and inflammatory changes over time. This study sets a precedent for utilizing MSI in retinal pathophysiology research, paving the way for future investigations into the metabolic underpinnings of retinal diseases.

While MSI offers unprecedented spatial resolution and sensitivity in lipid analysis, there are specific limitations to this technique. The ionization efficiency of different lipid species can vary, leading to potential underrepresentation of certain lipids. Additionally, the quantitative accuracy of MSI can be influenced by matrix effects and signal suppression, which may affect the detection of low-abundance lipids (46). Despite these limitations, the spatial information provided by MSI significantly enhances our understanding of lipid dysregulation in the PEX1-G844D mouse model.

## Conclusion

Our results highlight the importance of RPE in understanding *Pex1*-associated retinal dysfunction. Early lipid alterations and the subsequent inflammatory and structural defects suggest that the RPE plays a key role in the progression of retinopathy. An important finding in this study is the association of lipid changes and inflammation, though causality remains unknown. These findings pave the way for future research aimed at identifying which lipids drive the RD, and whether the underlying pathways could be novel or complementary therapeutic targets in the early stages of disease. Although we focus here on lipid profiles, peroxisome functions are broad and include cellular redox maintenance and immune modulation (47, 48). Equally, deficiencies in these functions and their contribution to the RD phenotype warrant further investigation.

## Data availability

Retinal tissue images used for structural phenotyping (cell number/density and inflammatory cell quantification) are located at the RI-MUHC. Raw data from MSI, MS/MS, and LC/MS/MS analyses are located at Université de Montréal, and the RI-MUHC.

## Supplemental data

This article contains supplemental data.

## Conflict of interest

The authors declare that they have no conflicts of interest with the contents of this article.
