# Supplementary material for: Spatial characterization of RPE structure and lipids in the PEX1-p.Gly844Asp mouse model for Zellweger spectrum disorder
Source: J Lipid Res. 2025 Mar 7;66(4):100771. doi: 10.1016/j.jlr.2025.100771 (PMC11999432; doi:10.1016/j.jlr.2025.100771)
Supplement: Supplemental tables [file mmc1.pdf]

Supporting Information

Supplemental Table S1. MSI lipid identification and relative levels in WT vs PEX1-G844D RPE flatmounts at 1 month of age

Lipid species (molecular species)	m/z	Control 1M	PEX1-G844D 1M	*P* value
**Negative ion mode - Dual-polarity MALDI MSI**				
PC (18:1_16:0)	744,5508	146 ± 7	108 ± 4	0,0013
PE (18:2_16:0)	714,5043	17 ± 36	39 ± 5	0,0019
PE (16:0_20:4)	738,5032	88 ± 36	164 ± 11	0,0253
PE 38:5	764,5178	35 ± 8	52 ± 2	0,03
PE (22:6_16:0)	762,5079	92 ± 16	51 ± 11	0,0202
PE (22:6_18:0)	790,5338	150 ± 30	68 ± 18	0,0209
PE 42:6	818,5656	18 ± 3	12 ± 1	0,0268
PI (18:0_18:2)	861,5469	15 ± 1	26 ± 0,8	0,0002
PI 36:3	859,5267	14 ± 2	21 ± 2	0,0114
PI (16:0_20:4)	857,5126	43 ± 12	58 ± 3	0,1125
PI (18:0_20:4)	885,5433	410 ± 120	189 ± 20	0,0365
PI 38:5	883,5286	22 ± 4	31 ± 1	0,0309
PI (18:0_22:6)	909,5432	23 ± 4	16 ± 2	0,0426
PA 38:2	763,5005	36 ± 6	22 ± 4	0,0345
PA 40:6	747,4947	33 ± 4	15 ± 1	0,0015
PS (18:0_20:4)	810,5246	24 ± 2	18 ± 1	0,0100
PG (22:6_22:6)	865,5079	15 ± 3	5,9 ± 0,8	0,0085
LPE O(34:0);O	742,5544	54 ± 20	68 ± 11	0,1885
LPE O(34:1);O	740,5370	121 ± 22	150 ± 22	0,0065
LPC O-32:3	698,5454	41 ± 5	29 ± 3	0,0275
PE O-36:5	722,5082	59 ± 8	29 ± 3	0,0038
PE O-38:5	750,5404	44 ± 4	19 ± 1	0,0005
PE O-40:7	774,5393	30 ± 2	14,3 ± 1,6	0,0003
PA O-36:1	687,5355	250 ± 11	220 ± 30	0,2177
PA O-42:1	771,6332	184 ± 21	140 ± 20	0,0573
PA O-42:2	769,6179	93 ± 20	61 ± 8	0,061
**Positive ion mode - Dual-polarity MALDI MSI**				
PC 34:2	780,5507	304 ± 18	386 ± 20	0,0063
PC 34:3	794,5072	30 ± 3	23 ± 0,9	0,012
PC 34:4	792,4935	51 ± 2	39 ± 2	0,0011
PC 36:0	812,6115	158 ± 8	199 ± 12	0,0083
PC 36:1	788,6188	212 ± 16	230 ± 14	0,1986
PC 36:2	786,6025	268 ± 23	330 ± 20	0,028
PC 36:3	822,5406	33 ± 2	29 ± 0,8	0,012
PC 36:4	782,5638	970 ± 140	970 ± 70	0,9666
PC 36:6	778,5376	80 ± 7	68 ± 0,6	0,0475
PC 38:4	810,6014	386 ± 30	388 ± 6	0,9288
PC 38:5	830,5611	113 ± 23	128 ± 9	0,367
PC 38:6	806,5701	540 ± 90	411 ± 34	0,0758
PC (16:1_22:6)	804,5543	260 ± 70	316 ± 14	0,2585
PC 40:4	860,6132	6 ± 0.4	9,4 ± 0,6	0,0011
PC 40:5	858,5996	14 ± 0.5	20,6 ± 0,6	0,0001
PC (18:0_22:6)	856,5827	44 ± 4	34 ± 3	0,024
PC 40:7	832,5851	25 ± 2	30 ± 3	0,081
PC 40:9	828,5374	173 ± 17	135 ± 12	0,0343
PC 42:5	886,6254	2,3 ± 0,2	3,2 ± 0,3	0,0093
PC 42:6	884,6104	3,4 ± 0,2	4,1 ± 0,4	0,0424
PC 42:10	892,5313	1,8 ± 0,1	2,3 ± 0,2	0,0127
PE 38:4	790,5311	194 ± 12	198 ± 5	0,6050
PA 40:5	773,5083	52 ± 5	52 ± 4	0,9827
SM 34:1;O2	725,5544	178 ± 50	158 ± 22	0,5807
SM 36:1;O2	731,6064	19 ± 2	29 ± 2	0,0017
SM 42:1 (d18:0_24:1)	837,6820	29 ± 2	38 ± 2	0,0045
LPC 16:0	496,3370	82 ± 19	77 ± 15	0,7155
LPC 18:0	524,3705	27 ± 10	19 ± 5	0,2629
LPC 18:1	522,3534	11 ± 2	7,6 ± 1,1	0,0553
LPC 18:2	520,3380	16 ± 3	17 ± 3	0,6315
LPC 20:4	544,3393	27 ± 7	20 ± 2	0,1621
LPC 22:6	568,3373	25 ± 3	12 ± 1	0,0031
LPC 26:6;O	678.3544	245 ± 43	215 ± 35	0,3957
**Silver-assisted LDI MSI**				
FA 16:0	363,1436	17 ± 4	16,1 ± 0,7	0,767
FA 18:0	391,1734	38 ± 5	25,6 ± 0,7	0,0118
FA 18:2	387,1436	14 ± 2	18,8 ± 2,1	0,0733
FA 20:4	411,1449	42 ± 3	35 ± 4	0,0655
FA 22:0;O	463,2328	13 ± 2	3,8 ± 1,0	0,001
FA 22:1;O4	509,2042	19 ± 6	5,4 ± 0,6	0,0177
FA 22:6	435,1444	80 ± 8	25,2 ± 7,5	0,001
FA 34:5	605,3409	0,67 ± 0,06	7,4 ± 1,2	0,0007
FA 32:5	577,3145	0,51 ± 0,05	4,8 ± 1,1	0,0023
FA 34:6	603,3368	0,66 ± 0,1	4,0 ± 0,9	0,0033
FA 36:6	631,3602	0,52 ± 0,03	2,0 ± 0,4	0,0035
ST 24:6;O2	457,1272	14 ± 2	3,4 ± 1,9	0,0031
ST 27:1;O	493,2595	14 ± 3	11 ± 0,7	0,1579

Supplemental Table S2. Lipid identification using MS/MS in mouse RPE tissue

m/z measured	Identification	Ion	Mass Error (ppm)	Supporting fragment ions
**Negative ion mode**				
714.5079	PE(16:0_18:2)	$[M-H]^-$	5.04	255, 279, 434, 452
722.5130	PE(P-16:0_20:4)	$[M-H]^-$	6.64	303
738.5079	PE(16:0_20:4)	$[M-H]^-$	6.36	255, 259, 303, 434, 452
744.5508	PC(16:0_18:1)	$[M-CH_3]^-$	5.51	168, 255, 281, 480, 488, 506
762.5079	PE(16:0_22:6)	$[M-H]^-$	6.95	255, 327, 452, 506
790.5392	PE(18:0_22:6)	$[M-H]^-$	6.83	283, 327, 462, 480, 506, 524
810.5246	PS(18:0_20:4)	$[M-H]^-$	5.55	283, 303, 419, 437, 439, 723
857.5186	PI(16:0_20:4)	$[M-H]^-$	7.00	153, 223, 241, 255, 297, 303, 315, 391,553, 571
861.5499	PI(18:0_18:2)	$[M-H]^-$	3.48	241, 279, 283, 581
865.5025	PG(22:6/22:6)	$[M-H]^-$	-6.24	283, 327
885.5499	PI(18:0_20:4)	$[M-H]^-$	7.45	223, 241, 259, 283, 303, 419, 440, 582, 603
909.5499	PI(18:0_22:6)	$[M-H]^-$	7.37	223, 241, 259, 283, 297, 315, 327, 419, 437, 481, 581, 599, 625, 747
**Positive ion mode**				
524.3705	LPC 18:0	$[M+H]^+$	2.10	184, 258, 341, 465, 506
568.3394	LPC 22:6	$[M+H]^+$	4.40	147, 184, 509
678.3544	LPC 26:6;O	$[M+K]^+$	-1.77	184
725.5544	SM 34:1;O_2	$[M+Na]^+$	3.31	147, 666
758.5714	PC 34:2	$[M+H]^+$	-2.64	124, 147, 184, 699
773.5083	PA 40:5	$[M+K]^+$	1.29	147, 649, 755
780.5507	PC 34:2	$[M+Na]^+$	0.90	147
790.5711	PE 38:4	$[M+Na]^+$	5.82	649
804.5543	PC 38:7	$[M+H]^+$	-0.62	147, 745
806.5701	PC 38:6	$[M+H]^+$	-0.87	184, 623, 747
820.5203	PC 36:4	$[M+K]^+$	6.09	124, 761
828.5539	PC 40:9	$[M+H]^+$	1.33	184, 645, 769
834.6010	PC 40:6	$[M+H]^+$	-0.36	147, 184, 651, 775
844.5279	PC 38:6	$[M+K]^+$	-3.08	184, 661, 785
856.5854	PC 40:6	$[M+Na]^+$	-1.64	147, 184, 673, 797
**Fatty acids**				
363.1436	FA 16:0	$[M+Ag^{107}]^+$	3.03	317, 345
387.1436	FA 18:2	$[M+Ag^{107}]^+$	2.84	221, 259, 285, 369
391.1734	FA 18:0	$[M+Ag^{107}]^+$	6.65	345, 373
411.1449	FA 20:4	$[M+Ag^{107}]^+$	-0.49	257, 273, 297, 313, 365, 393
435.1444	DHA FA 22:6	$[M+Ag^{107}]^+$	0.69	215, 255, 258, 299, 391, 417
457.1272	ST 24:6;O_2	$[M+Ag^{107}]^+$	3.94	439
463.2328	FA 22:0;O	$[M+Ag^{107}]^+$	1.51	445
493.2595	Cholesterol	$[M+Ag^{107}]^+$	-0.41	367, 437, 475
509.2042	FA 22:1;O_4	$[M+Ag^{107}]^+$	-3.41	491
577.3145	FA 32:5	$[M+Ag^{107}]^+$	3.98	239, 559
605.3409	FA 34:5	$[M+Ag^{107}]^+$	11.89	324, 587

Supplemental Table S3. Comparison of relative lipid levels in dorsal *vs* ventral pole of WT or PEX1-G844D RPE flatmounts analysed by MSI at 1 month of age

Lipid species (molecular species)	Ventral control 1M	Dorsal control 1M	P value	Ventral PEX1-G844D 1M	Dorsal PEX1-G844D 1M	P value
**Negative ion mode – Dual-polarity MALDI MSI**						
PC (16:0_18:1)	163 ± 18	147 ± 3	0,2177	125 ± 5	106 ± 11	0,0587
PE (18:2/16:0)	20 ± 1	18 ± 1	0,1721	44 ± 6	43 ± 6	0,9153
PE (16:0_20:4)	99 ± 37	99 ± 34	0,9957	203 ± 8	169 ± 27	0,1086
PE 38:5	38 ± 9	39 ± 7	0,9104	58 ± 2	55 ± 6	0,3990
PE (22:6/16:0)	107 ± 15	93 ± 12	0,3044	56 ± 11	56 ± 15	0,9896
PE (22:6/18:0)	171 ± 34	143 ± 20	0,3218	74 ± 20	76 ± 23	0,9457
PE 42:6	21 ± 3	18 ± 1	0,2094	13 ± 1	12 ± 2	0,5435
PI (18:0/18:2)	17 ± 1	15 ± 1	0,1094	30 ± 2	29 ± 3	0,6370
PI 36:3	14 ± 3	14 ± 2	0,8151	23 ± 1	22 ± 2	0,6101
PI (16:0/20:4)	50 ± 15	44 ± 9	0,6117	71 ± 7	58 ± 1	0,0461
PI (18:0/20:4)	485 ± 118	424 ± 118	0,5979	232 ± 38	191 ± 38	0,2589
PI 38:5	26 ± 4	23 ± 4	0,5607	35 ± 1	35 ± 4	0,8839
PI (18:0/22:6)	30 ± 3	23 ± 4	0,1240	18 ± 1	17 ± 3	0,5008
PA 38:2	42 ± 6	37 ± 5	0,3271	25 ± 5	24 ± 5	0,8837
PA 40:6	38 ± 3	34 ± 3	0,1579	16 ± 2	16 ± 2	0,8172
PS (18:0_20:4)	26 ± 3	25 ± 2	0,5984	19 ± 1	18 ± 2	0,6978
PG (22:6/22:6)	17 ± 3	15 ± 3	0,4255	7 ± 1	6 ± 1	0,7474
LPE O(34:1);O	14 ± 4	14 ± 3	0,8664	27 ± 1	24 ± 3	0,1294
LPE O(34:0);O	145 ± 25	123 ± 19	0,3327	187 ± 30	150 ± 28	0,1978
LPC O-32:3	48 ± 4	41 ±5	0,2176	37 ± 4	28 ± 4	0,0796
PE O-36:5	65 ± 9	63 ± 8	0,8131	33 ± 4	28 ± 2	0,1844
PE O-38:5	47 ± 6	45 ± 3	0,6366	20 ± 1	20 ± 2	0,9303
PE O-40:7	34 ± 2	30 ± 2	0,0699	16 ± 2	14 ± 1	0,2641
PA O-36:1	284 ± 33	247 ± 2	0,1225	231,12	234,87	0,9163
PA O-42:1	222 ± 8	187 ± 27	0,1435	181 ± 18	138 ± 24	0,0678
PA O-42:2	113 ± 18	95 ± 19	0,3684	80 ± 11	57 ± 10	0,0574
**Positive ion mode - Dual-polarity MALDI MSI**						
PC 34:2	308 ± 15	320 ± 22	0,5434	443 ± 4	371 ± 39	0,0325
PC 34:3	29 ± 3	30 ± 2	0,5490	22 ± 1	22 ± 2	0,8322
PC 34:4	49 ± 5	51 ± 2	0,5856	38 ± 1	39 ± 3	0,4193
PC 36:0	144 ± 9	164 ± 9	0,0775	179 ± 15	208 ± 12	0,0534
PC 36:1	212 ± 21	212 ± 12	0,9944	220 ± 12	237 ± 19	0,2646
PC 36:2	284 ± 25	274 ± 19	0,6670	340 ± 29	341 ± 15	0,9773
PC 36:3	34 ± 1	37 ± 3	0,3264	30 ± 2	31 ± 2	0,4504
PC 36:4	1007 ± 176	997 ± 114	0,9398	1036 ±127	958 ± 34	0,3618
PC 36:6	86 ± 9	84 ± 5	0,7907	78 ± 2	68 ± 5	0,0305
PC 38:4	38 ± 24	397 ± 31	0,5808	362 ± 19	414 ± 12	0,0165
PC 38:5	116 ± 23	127 ± 24	0,6371	128 ± 11	150 ± 13	0,0940
PC 38:6	593 ± 91	575 ± 77	0,8177	449 ± 47	440 ± 42	0,8217
PC (16:1_22:6)	256 ± 64	290 ± 64	0,5985	331 ± 19	326 ± 18	0,7685
PC 40:4	5,8 ± 0,1	6,6 ± 0,6	0,1636	8,4 ± 0,2	11 ± 1	0,0090
PC 40:5	13 ± 1	15 ± 2	0,2532	18 ± 1	25 ± 2	0,0037
PC (18:0/22:6)	41 ± 4	47 ± 7	0,3512	29 ± 3	41 ± 2	0,0082
PC 40:7	143 ± 21	163 ± 28	0,4285	144 ± 10	181 ±2	0,0030
PC 42:5	2,4 ± 0,2	2,3 ± 0,1	0,5838	3,1 ± 0,2	3,8 ± 0,3	0,0400
PC 42:6	3,4 ± 0,3	3,5 ± 0,2	0,7226	3,8 ± 0,3	4,8 ± 0,4	0,0293
PC 42:10	2,0 ± 0,2	2,1 ± 0,1	0,6844	2,5 ± 0,1	3,0 ± 0,1	0,0102
PE 38:4	192 ± 21	197 ± 10	0,7198	193 ± 8	207 ± 12	0,1638
PA 40:5	56 ± 3	55 ± 8	0,8914	58 ± 3	55 ± 5	0,4557
SM 36:1;O2	21 ± 1	19 ± 1	0,0666	32 ± 3	30 ± 2	0,2443
SM 42:1	27 ± 4	31 ± 5	0,4140	32 ± 1	43 ± 2	0,0016
SM 34:1;O2	252 ± 59	214 ± 38	0,4283	194 ± 58	206 ± 25	0,7505
LPC 16:0	88 ± 20	80 ± 19	0,6901	68 ± 6	93 ± 29	0,2157
LPC 18:0	31 ± 12	25 ± 9	0,5971	14 ± 1	26 ± 11	0,1447
LPC 18:1	11 ± 1	10 ± 1	0,5295	7 ± 1	8 ± 3	0,3640
LPC 18:2	19 ± 4	15 ± 3	0,3411	20 ± 5	18 ± 3	0,6518
LPC 20:4	32 ± 10	26 ± 4	0,4099	22 ± 4	22 ± 2	0,9229
LPC 22:6	29 ± 3	22 ± 2	0,0421	12 ± 1	13 ± 2	0,4533
LPC 26:6;O	252 ± 59	214 ± 38	0,4283	194 ± 58	206 ± 25	0,7505
**Silver-assisted LDI MSI**						
FA 16:0	20 ± 6	15 ± 1	0,2206	18 ± 3	17 ±2	0,6698
FA 18:0	44 ± 8	33 ± 2	0,0724	24 ± 4	27 ± 2	0,3839
FA 18:2	17 ± 3	13 ± 1	0,0940	19 ± 4	21 ± 2	0,6266
FA 20:4	49 ± 6	39 ± 4	0,1074	33 ± 8	39 ± 4	0,3361
FA 22:0;O	8 ± 1	5 ± 0,2	0,0168	6 ± 1	5 ± 1	0,1533
FA 22:1;O4	26 ± 10	16 ± 1	0,1872	7 ± 2	5 ± 1	0,1444
FA 22:6	107 ± 10	72 ± 12	0,0280	32 ± 15	24 ± 8	0,4701
FA 32:5	0,6 ± 0,1	0,4 ± 0	0,0313	5 ± 2	6 ± 1	0,4975
FA 34:5	0,8 ± 0,1	0,5 ± 0	0,0251	7 ± 2	10 ± 1	0,1891
FA 34:6	0,7 ± 0,1	0,5 ± 0	0,1470	4 ± 1	5 ± 0	0,509
FA 36:6	0,6 ± 0,1	0,4 ± 0	0,0401	2 ± 0	2 ± 0	0,3184
ST 24:6;O2	19 ± 2	13 ± 4	0,1271	5 ± 3	3 ± 2	0,5321
ST 27:1;O	161 ± 56	137 ± 14	0,5212	130 ± 8	153 ± 15	0,0778

Supplemental Table S4. MSI lipid identification and relative levels in WT vs PEX1-G844D RPE flatmounts at 3 months of age

Lipid species (molecular species)	m/z	Control 3M	PEX1-G844D 3M	*P* value
**Negative ion mode - Dual-polarity MALDI MSI**				
PC (18:1_16:0)	744,5508	90 ± 23	68 ± 14	0,2236
PE (18:2_16:0)	714,5043	13 ± 3	26 ± 4	0,0097
PE (16:0_20:4)	738,5032	114 ± 29	150 ± 20	0,1772
PE 38:5	764,5178	39 ± 7	64 ± 8	0,0169
PE (22:6_16:0)	762,5079	145 ± 39	130 ± 6	0,5429
PE (22:6_18:0)	790,5338	179 ± 63	210 ± 28	0,4517
PE 42:6	818,5656	17 ± 6	19 ± 2	0,7261
PI (18:0_18:2)	861,5469	15 ± 3	23 ± 3	0,0537
PI 36:3	859,5267	16 ± 2	20 ± 4	0,1871
PI (16:0_20:4)	857,5126	68 ± 19	73 ± 12	0,7047
PI (18:0_20:4)	885,5433	493 ± 108	290 ± 21	0,0332
PI 38:5	883,5286	19 ± 3	23 ± 3	0,1222
PI (18:0_22:6)	909,5432	38 ± 7	22 ± 5	0,0268
PA 38:2	763,5005	59 ± 16	53 ± 2	0,5897
PA 40:6	747,4947	48 ± 10	21 ± 1	0,0103
PS (18:0_20:4)	810,5246	45 ± 7	26 ± 3	0,0138
PG (22:6_22:6)	865,5079	3,6 ± 0,4	4.4 ± 0.4	0,0718
LPE O(34:0);O	742,5544	87 ± 28	120 ± 23	0,2023
LPE O(34:1);O	740,5370	12 ± 3	19 ± 3	0,04
LPC O-32:3	698,5454	21,5 ± 3,4	17 ± 2	0,1237
PE O-36:5	722,5082	60,6 ± 8	28.5 ± 5.5	0,0046
PE O-38:5	750,5404	60,6 ± 8	25.6 ± 5.4	0,0011
PE O-40:7	774,5393	46 ± 15	21.4 ± 1.8	0,0467
PA O-36:1	687,5355	148 ± 29	106 ± 8	0,078
PA O-42:1	771,6332	96 ± 41	51 ± 9	0,1329
PA O-42:2	769,6179	54 ± 22	35 ± 5	0,2042
**Positive ion mode - Dual-polarity MALDI MSI**				
PC 34:2	780,5507	213 ± 24	340 ± 60	0,0257
PC 34:3	794,5072	32,6 ± 2	21.7 ± 1.2	0,0012
PC 34:4	792,4935	46,6 ± 1,6	33 ± 3	0,0034
PC 36:0	812,6115	136 ± 11	165 ± 17	0,0688
PC 36:1	788,6188	229 ± 17	160 ± 19	0,0085
PC 36:2	786,6025	194 ± 38	270 ± 32	0,0555
PC 36:3	822,5406	26,3 ± 1,3	31 ± 4	0,12
PC 36:4	782,5638	745 ± 33	950 ± 80	0,0178
PC 36:6	778,5376	92 ± 9	68 ± 8	0,0239
PC 38:4	810,6014	355 ± 25	370 ± 57	0,741
PC 38:5	830,5611	69 ± 20	120 ± 11	0,0191
PC 38:6	806,5701	354 ± 80	430 ± 52	0,2687
PC (16:1_22:6)	804,5543	169 ± 20	340 ± 27	0,0009
PC 40:4	860,6132	7,8 ± 0,7	11.7 ± 0.1	0,0006
PC 40:5	858,5996	14,7 ± 1,8	25 ± 2	0,0033
PC (18:0_22:6)	856,5827	46,6 ± 6,8	45 ± 8	0,8079
PC 40:7	832,5851	115 ± 15	170 ± 25	0,0379
PC 40:9	828,5374	122 ± 21	153 ± 17	0,1121
PC 42:5	886,6254	3,0 ± 0,4	4 ± 0.3	0,0272
PC 42:6	884,6104	4,6 ± 0,3	5.7 ± 0.4	0,0211
PC 42:10	892,5313	2,5 ± 0,3	3.6 ± 0.5	0,0295
PE 38:4	790,5311	208 ± 11	156 ± 23	0,0240
PA 40:5	773,5083	31 ± 2	63 ± 12	0,0105
SM 34:1;O2	725,5544	261 ± 54	124 ± 12	0,0127
SM 36:1;O2	731,6064	16,4 ± 1,9	19 ± 4	0,3337
SM 42:1 (d18:0_24:1)	837,6820	56 ± 3	56 ± 2	0,8891
LPC 16:0	496,3370	84 ± 14	72 ± 2	0,2089
LPC 18:0	524,3705	58 ± 21	20 ± 2	0,0337
LPC 18:1	522,3534	21,8 ± 2,3	6.6 ± 1.2	0,0006
LPC 18:2	520,3380	16 ± 8	11 ± 6	0,4188
LPC 20:4	544,3393	18 ± 8	10 ± 3	0,1963
LPC 22:6	568,3373	23 ± 5	9.3 ± 1.1	0,0091
LPC 26:6;O	678.3544	393 ± 68	160 ± 13	0,0043
**Silver-assisted LDI MSI**				
FA 16:0	363,1436	22,4 ± 3,1	14 ± 2	0,0195
FA 18:0	391,1734	45 ± 11	21 ± 3	0,0224
FA 18:2	387,1436	17,6 ± 1,9	16 ± 4	0,5555
FA 20:4	411,1449	44,3 ± 3,7	25 ± 6	0,0084
FA 22:0;O	463,2328	8,5 ± 0,8	3.8 ± 0.5	0,0012
FA 22:1;O4	509,2042	23,3 ± 2,9	4.1 ± 0.4	0,0003
FA 22:6	435,1444	100 ± 21	12.5 ± 1.2	0,0018
FA 34:5	605,3409	0,9 ± 0,1	31 ± 8	0,0024
FA 32:5	577,3145	0,7 ± 0,1	14.6 ± 3.8	0,0032
FA 34:6	603,3368	0,9 ± 0,1	15.3 ± 2.3	0,0004
FA 36:6	631,3602	0,6 ± 0,1	5.3 ± 1.8	0,0107
ST 24:6;O2	457,1272	12,5 ± 3	1.6 ± 0.2	0,0035
ST 27:1;O	493,2595	214 ± 49	170 ± 18	0,1907

Supplemental Table S5. Comparison of relative lipid levels in dorsal *vs* ventral pole of WT or PEX1-G844D RPE flatmounts analysed by MSI at 3 months of age

Lipid species (molecular species)	Ventral control 3M	Dorsal control 3M	P value	Ventral PEX1-G844D 3M	Dorsal PEX1-G844D 3M	P value
**Negative ion mode – Dual-polarity MALDI MSI**						
PC (16:0_18:1)	99 ± 24	98 ± 25	0,9685	61 ± 14	81 ± 15	0,1708
PE (18:2/16:0)	13 ± 3	15 ± 5	0,5412	16 ± 2	50 ± 9	0,0032
PE (16:0_20:4)	128 ± 42	153 ± 21	0,4047	64 ± 10	364 ± 49	0,0004
PE 38:5	41 ± 7	46 ± 8	0,5375	60 ± 8	82 ± 10	0,0382
PE (22:6/16:0)	169 ± 35	178 ± 44	0,8102	151 ± 4	150 ± 20	0,8980
PE (22:6/18:0)	220 ± 67	202 ± 56	0,7425	406 ± 57	130 ± 17	0,0013
PE 42:6	21 ± 6	19 ± 7	0,6933	27 ± 5	16 ± 2	0,0184
PI (18:0/18:2)	16 ± 3	18 ± 8	0,7611	18 ± 2	35 ± 7	0,0159
PI 36:3	16 ± 2	18 ± 4	0,5093	18 ± 3	29 ± 9	0,1124
PI (16:0/20:4)	71 ± 19	87 ± 25	0,4028	57 ± 11	128 ± 29	0,0170
PI (18:0/20:4)	524 ± 115	626 ± 114	0,3365	305 ± 51	350 ± 29	0,2497
PI 38:5	19 ± 2	22 ± 5	0,3397	21 ± 1	33 ± 8	0,0505
PI (18:0/22:6)	42 ± 3	53 ± 12	0,2055	23 ± 5	29 ± 7	0,3127
PA 38:2	69 ± 13	70 ± 18	0,9286	61 ± 2	60 ± 6	0,8443
PA 40:6	57 ± 12	54 ± 7	0,7398	25 ± 1	20 ± 1	0,0050
PS (18:0_20:4)	68 ± 19	68 ± 9	0,9705	20 ± 2	51 ± 4	0,0003
PG (22:6/22:6)	53 ± 10	53 ± 17	0,9458	17 ± 5	10 ± 1	0,0681
LPE O(34:1);O	13 ± 3	14 ± 3	0,8008	12 ± 2	35 ± 5	0,0018
LPE O(34:0);O	98 ± 26	102 ± 36	0,8843	101 ± 18	181 ± 38	0,0311
LPC O-32:3	24 ± 4	25 ±4	0,5549	15 ± 2	22 ± 3	0,0324
PE O-36:5	64 ± 11	66 ± 10	0,8115	24 ± 4	36 ± 7	0,0670
PE O-38:5	59 ± 7	63 ± 0	0,3795	24 ± 5	27 ± 6	0,5487
PE O-40:7	53 ± 18	51 ± 14	0,9136	23 ± 1	22 ± 3	0,4699
PA O-36:1	165 ± 29	157 ± 34	0,7804	102 ± 9	115 ± 7	0,1283
PA O-42:1	112 ± 52	115 ± 41	0,9431	39 ± 7	78 ± 13	0,0097
PA O-42:2	64 ± 24	67 ± 24	0,8920	30 ± 3	53 ± 8	0,0111
**Positive ion mode – Dual-polarity MALDI MSI**						
PC 34:2	235 ± 34	215 ± 24	0,4444	394 ± 76	333 ± 56	0,3293
PC 34:3	34 ± 3	32 ± 1	0,5354	21 ± 1	21 ± 4	0,8979
PC 34:4	47 ± 3	49 ± 1	0,3172	35 ± 4	31 ± 4	0,2593
PC 36:0	138 ± 14	135 ± 8	0,7296	174 ± 28	161 ± 26	0,5819
PC 36:1	234 ± 12	236 ± 25	0,9454	175 ± 17	146 ± 17	0,1150
PC 36:2	216 ± 56	200 ± 40	0,7108	294 ± 28	295 ± 61	0,9651
PC 36:3	29 ± 2	27 ± 2	0,3075	32 ± 4	34 ± 11	0,7423
PC 36:4	774 ± 52	754 ± 51	0,6568	1031 ± 33	1017 ± 233	0,9261
PC 36:6	100 ± 9	92 ± 10	0,3889	74 ± 11	63 ± 21	0,4939
PC 38:4	358 ± 34	367 ± 27	0,7378	364 ± 66	421 ± 112	0,4948
PC 38:5	80 ± 26	76 ± 27	0,8686	135 ± 14	126 ± 14	0,4965
PC 38:6	403 ± 103	390 ± 124	0,9017	467 ± 42	477 ± 91	0,8624
PC (16:1_22:6)	184 ± 30	171 ± 25	0,5913	365 ± 26	371 ± 81	0,9014
PC 40:4	7 ± 1	8 ± 1	0,5759	11 ± 1	12 ± 1	0,2692
PC 40:5	15 ±3	16 ± 2	0,5573	24 ± 5	28 ± 1	0,2106
PC (18:0_22:6)	46 ± 7	53 ± 12	0,4125	41 ± 13	53 ± 9	0,2698
PC 40:7	119 ± 22	119 ± 17	0,9852	157 ± 38	194 ± 46	0,3431
PC 40:9	132 ± 24	135 ± 35	0,9160	161 ± 23	168 ± 15	0,6619
PC 42:5	2,8 ± 0,5	3 ± 0,3	0,7102	4 ± 0	4 ± 1	0,7245
PC 42:6	4,3 ± 0,4	4,6 ± 0,2	0,4285	5 ± 0	6 ± 1	0,9361
PC 42:10	2,4 ± 0,2	2,6 ± 0,3	0,5438	3,4 ± 0,4	4 ± 1	0,4236
PE 38:4	210 ± 8	215 ± 18	0,6731	178 ± 23	142 ± 23	0,1270
PA 40:5	35 ± 3	32 ± 4	0,3773	67 ± 13	74 ± 28	0,7148
SM 36:1;O2	19 ± 3	16 ± 1	0,3047	24 ± 3	17 ± 5	0,1250
SM 42:1	54 ± 5	61 ± 3	0,1129	60 ± 4	51 ± 5	0,0514
SM 34:1;O2	264 ± 77	263 ± 76	0,9971	106 ± 13	125 ± 66	0,6398
LPC 16:0	87 ± 18	89 ± 18	0,8980	72 ± 16	80 ± 18	0,5740
LPC 18:0	64 ± 21	64 ± 26	0,9953	18 ± 7	24 ± 3	0,286
LPC 18:1	23 ± 3	23 ± 3	0,8286	7 ± 1	6 ± 2	0,4992
LPC 18:2	20 ± 13	16 ± 7	0,6670	15 ± 5	10 ± 8	0,3996
LPC 20:4	21 ± 13	19 ± 6	0,8045	13 ± 2	10 ± 5	0,3381
LPC 22:6	26 ± 8	25 ± 3	0,9740	9 ± 1	9 ± 3	0,8992
LPC 26:6;O	420 ± 92	395 ± 85	0,7508	152 ± 2	153 ± 57	0,9842
Silver-assisted LDI MSI						
FA 16:0	24 ± 2	21 ± 2	0,1805	17 ± 4	12 ± 1	0,0727
FA 18:0	52 ± 15	42 ± 11	0,3974	24 ± 4	18 ± 3	0,1516
FA 18:2	19 ± 1	17 ± 2	0,2239	19 ± 4	14 ± 4	0,2900
FA 20:4	51 ± 7	41 ± 1	0,0836	29 ± 8	22 ± 4	0,2446
FA 22:0;O	10 ± 1	8 ± 0	0,0693	5 ± 1	2,8 ± 0,3	0,0258
FA 22:1;O4	33 ± 4	23 ± 6	0,0639	5 ± 1	3,3 ± 0,5	0,0258
FA 22:6	149 ± 44	90 ± 14	0,0887	13 ± 2	13 ± 3	0,9851
FA 32:5	0,9 ± 0,2	0,6 ± 0	0,0464	22 ± 8	12 ± 5	0,1405
FA 34:5	1,2 ± 0,2	0,7 ± 0,1	0,0392	32 ± 13	35 ± 10	0,8015
FA 34:6	1,1 ± 0,2	0,8 ± 0,1	0,0942	18 ± 4	16 ± 7	0,7650
FA 36:6	0,7 ± 0	0,5 ± 0	0,0084	4 ± 2	7 ± 2	0,1792
ST 24:6;O2	17 ± 5	12 ± 3	0,2259	1,6 ± 0,1	1,6 ± 0,4	0,9719
ST 27:1;O	226 ±55	214 ± 55	0,8016	186 ± 26	139 ± 15	0,0567
